# Developing content for a process-of-care checklist for use in intensive care units: a dual-method approach to establishing construct validity

**DOI:** 10.1186/1472-6963-13-380

**Published:** 2013-10-03

**Authors:** Karena M Conroy, Doug Elliott, Anthony R Burrell

**Affiliations:** 1 Faculty of Health, University of Technology, Sydney, PO Box 123, Broadway, NSW 2007, Australia; 2 Intensive Care Co-ordination & Monitoring Unit, NSW Agency for Clinical Innovation, Chatswood, Australia; 3 NSW Clinical Excellence Commission, Locked Bag A4062, Sydney South, NSW 1235, Australia

**Keywords:** Checklists, Construct validity, Delphi technique, Healthcare quality improvement, Patient safety, Critical care

## Abstract

**Background:**

In the intensive care unit (ICU), checklists can be used to support the delivery of quality and consistent clinical care. While studies have reported important benefits for clinical checklists in this context, lack of formal validity testing in the literature prompted the study aim; to develop relevant ‘process-of-care’ checklist statements, using rigorously applied and reported methods that were clear, concise and reflective of the current evidence base. These statements will be sufficiently instructive for use by physicians during ICU clinical rounds.

**Methods:**

A dual-method approach was utilized; semi-structured interviews with local clinicians; and rounds of surveys to an expert Delphi panel. The interviews helped determine checklist item inclusion/exclusion prior to the first round Delphi survey. The panel for the modified-Delphi technique consisted of local intensivists and a state-wide ICU quality committee. Minimum standards for consensus agreement were set prior to the distribution of questionnaires, and rounds of surveys continued until consensus was achieved.

**Results:**

A number of important issues such as overlap with other initiatives were identified in interviews with clinicians and integrated into the Delphi questionnaire, but no additional checklist items were suggested, demonstrating adequate checklist coverage sourced from the literature. These items were verified by local clinicians as being relevant to ICU and important elements of care that required checking during ward rounds. Two rounds of Delphi surveys were required to reach consensus on nine checklist statements: nutrition, pain management, sedation, deep vein thrombosis and stress ulcer prevention, head-of-bed elevation, blood glucose levels, readiness to extubate, and medications.

**Conclusions:**

Statements were developed as the most clear, concise, evidence-informed and instructive statements for use during clinical rounds in an ICU. Initial evidence in support of the checklist’s construct validity was established prior to further prospective evaluation in the same ICU.

## Background

In pursuit of improving the safety and quality of care delivered to critically ill patients internationally, the use of practice improvement tools in intensive care units (ICUs) such as checklists is evident [1-4]. There is however a distinct lack of appropriate, effective and standardized methods noted in the literature for the development, design and testing of checklists in clinical settings. This gap highlights the need for rigorous validation work prior to checklist implementation [1,5]. General recommendations for the development of checklists include conducting a thorough literature review, evaluation of current practices, consideration of expert opinion and consensus, obtaining multidisciplinary input, and thorough validation of the checklist using an iterative approach [1,5].

In line with these recommendations, we conducted a comprehensive literature review that identified a number of processes of care (i.e. the practices involved in the delivery of care) suitable for the general ICU population including nutrition, pain, sedation, blood glucose, and medications management, deep vein thrombosis (DVT), stress ulcer, pressure sore, and catheter-related bloodstream infection prevention, weaning from mechanical ventilation and head-of-bed positioning for prevention of ventilator-associated pneumonia [3]. Evaluation of clinical practice via a point-prevalence study conducted in 50 Australian and New Zealand ICUs revealed deficiencies in the delivery of several aspects of care including assessment of nutritional goals, pain, sedation, and pressure area risk, head of bed elevation, weaning, and bowel management practices [6]. We also completed a criterion-related validation study where pilot checklist responses were compared with patient records [7]. While this study demonstrated support for the construct validity for eight specific processes of care (nutrition, weaning, BSL management, sit out of bed, bowel management, stress ulcer & DVT prevention), the following issues required further examination.

First, some items on the earlier version of the checklist [8] (i.e. head-of-bed elevation, assessment of sedation levels, reviewing medications) could not be assessed for their clinical utility due to lack of documentation during review of the medical records. Further evaluation was therefore needed to support the continued inclusion of these items in the checklist. Second, increased rigor in content development was highlighted. Checklist statements needed to be clear and concise enabling both comprehension and consistent interpretation [1]. Third, consideration of the local ICU context was identified as important to further checklist development and study design. After examining local ICU ward round practices and aspects of care that were ordered, managed and reviewed by physicians, local policies and guidelines, concurrent projects, and related work processes and procedures were also reviewed.

Establishing evidence in support of an instrument’s construct validity (the degree to which an instrument measures the construct it is intended to measure) requires examination of the sufficiency, relevance and clarity of content [9-11]. To begin developing validity for the checklist, it was essential to know that content reflected its intended purpose [11] and was relevant to the patient population and clinical setting, and useful for ICU physicians during morning rounds. This was partially addressed in our preliminary work [3,6,7], although further work was required for practice relevance at the local ICU level. Clarity was another component not yet formally evaluated in this development process.

A modified-Delphi technique is a suitable method to verify the content validity of a measure [9,12]. This approach involves collecting and organizing informed opinions from a panel of experts with specialized knowledge in the area being studied, purposely chosen to develop and refine the content of a specific measure during a series of consensus rounds [13]. Findings from a comprehensive literature review may be used to develop initial content for the first round of questionnaires [14]. Delphi techniques have been used to develop content for a variety of checklists for use in community nursing [13]; palliative care tools [15]; and a quality management model for integrated care [14].

To date, few studies have reported formal validity testing of checklists for use in clinical settings. e.g. [16-18] Three studies that utilized the Delphi technique focused on content development obtained via face validity with expert clinicians, but did not report a number of key methodological issues (see Table 1). As also noted in Table 1, two non-ICU studies comprehensively detailed the process of using Delphi techniques to develop content for a fall-risk checklist [13] and a simulation performance checklist to evaluate the performance of practicing anesthesiologists [19]. These two studies provided a model of how the Delphi technique can be used effectively in the development of checklist content. They did not however relate specifically to the development of a tool for measuring and ensuring the delivery of daily cares in an ICU.

**Table 1:** Studies utilizing the Delphi technique to develop content for checklists used in clinical settings

**Study**	**Sample**		**Purpose / Method**	Findings* / *Critique*
	**Setting**	**n / cohort**		
Huang, Lin & Lin. (Taiwan) [13]	College of Nursing	14 / 20 invited panel members accepted; 10 scholars in relevant fields of expertise, 4 clinical nurses.	● To develop content for a fall-risk checklist ● Framework presented to panel who were asked to review a 4-point Likert scale checklist (from strong agreement to strong disagreement), submit comments & provide revision suggestions ● Likert scale used to calculate content validity index (CVI) score for each item, rated along 3 dimensions i.e. content importance, appropriateness and discreteness ● Scoring calculation method detailed	● 70% of potential panel members accepted, 3 rounds required, completed over 4-month period ● Response rates: round 1, 78.5% (3 withdrew); 2, 91% (1 withdrew); 3, 100% ● Results of each round reported in summarized format ● Key suggestions & resulting refinements for each round provided ● Changes to domains and checklist processes documented ● CVI scores for each domain along the 3 dimensions and total score (range 0.84 – 1.00) in last review round provided ● *Information not provided: complete checklist, criteria for deleting items,* v*ariation in responses & scores to individual items (results summarized by domain)*
Morgan et al. (Canada) [19]	2 independent academic centers	5 anesthesiologists	● To develop a simulation performance checklist to evaluate performance of practicing anesthesiologists, using a computer-based Delphi technique ● Checklist items generated by participants after reading 2 pre-prepared scenarios, error weighting assigned to each item based on risk level ● Responses collated anonymously & emailed back to participants asking them to check off items to retain or delete & to (re)assign weightings ● Process repeated until no further items added, deleted or changes to weightings ● *A-priori* decision to delete responses endorsed by ≤ 20% respondents	● 100% response rate ● Required four rounds to reach consensus ● Participants generated 104 items for scenario 1 & 99 items for scenario 2 ● Final percentage weightings for checklist items provided ● *Small sample size* ● *Information not provided: variation in error weighting to individual items,* k*ey study timeframes e.g. time from survey distribution to response*
Hart & Owen. (Australia) [17]	Anesthesia Department at a tertiary hospital	Not reported - consultants with special interest in obstetric anesthesia	● To generate checklist items for use prior to commencing non-emergency Cesarean delivery under general anesthesia ● Participants contacted via email and remained anonymous to other participants ● Two questionnaires were circulated ● Two questionnaires were circulated	● Results of 2 questionnaires informed construction of checklist items ● Items were later divided into four sub-categories ● *Key information not reported: sample size; contents of questionnaires; response rates; how responses were used to inform 2nd round questionnaire & construct final checklist items e.g. not known whether pre-defined consensus methods were used, how checklist items were grouped & ordered*
Ursprung et al. (USA) [16]	20-bed tertiary care medical-surgical neonatal ICU	Not reported - experts in neonatology, pediatrics, health services research, systems engineering, infection control, advanced practice nursing	● To develop a patient safety audit checklist for PICUs ● Questions formatted into a checklist and refined iteratively by consensus ● Participants responses based on potential clinical impact of mistakes, system failures, perceived frequency ● Checklist reviewed and refined by physicians and nursing staff from study NICU to ensure relevance locally	● 36 audit questions representing a broad range of errors associated with NICU patient care generated ● Questions later divided into 2 categories ● *Information not reported: sample size and participant designations; contents of questionnaire; number of rounds required; method of obtaining consensus; how checklist items were further reviewed and refined for relevance by local PICU staff after consensus was reached; method of categorization*
Pronovost et al. (USA) [18]	13 adult medical & surgical ICUs in urban teaching & community hospitals	Interviews: 8 nurses & 5 ICU physicians Focus group: not reported	● Development and pilot testing of daily goals form ● Validity of measures: obtaining agreement from ICU physicians and quality experts who developed the measures; semi-structured interviews with nurses & physicians who piloted the measures ● Face validity: focus group of physicians and nurses from 13 participating ICUs	● Validity of measures: ICU physicians and quality experts unanimously agreed process measures addressed important aspects of ICU quality ● Focus group: participants believed measures ‘evaluated the domain of quality they intended to measure and identified important opportunities to improve quality’ [18], p.154 ● *Information not provided: sample sizes for development of measures and focus group; content for focus group discussion & semi-structured interviews; how qualitative data analyzed and interpreted*

Limitations evident in the literature on checklist content development highlight a gap in knowledge that needs to be addressed. The aim of this study was to develop the most relevant process-of-care checklist items that were clear, concise and descriptive statements for daily use by physicians during ward rounds in the ICU. These statements were to be generated using rigorously applied and reported methods, and be valid for use in a planned checklist intervention study. The specific research questions were:

1) What is the relevance and adequacy of the process measures identified from a literature review to the local ICU?

2) What are the most clear, concise and descriptive statements for use as checklist items?

## Methods

### Design

A dual-method approach was used for developing final content for the process-of-care checklist – local clinician interviews; and a modified-Delphi technique using an expert clinician panel. To explore the relevance and adequacy of the process measures local clinician input to checklist content was obtained via semi-structured one-on-one interviews with a purposive sample of clinical staff members at a tertiary level ICU of a university hospital. A modified-Delphi technique involving a wider purposive sample of experts was then constituted for refinement of consequent checklist items. This process enabled expert clinicians to develop consensus on clear and concise checklist statements. Given the previous development work (i.e. literature review [3], point prevalence study [6], and criterion-related checklist validation), 2–3 Delphi rounds were anticipated to reach consensus.

### Participants

**Figure 1:**
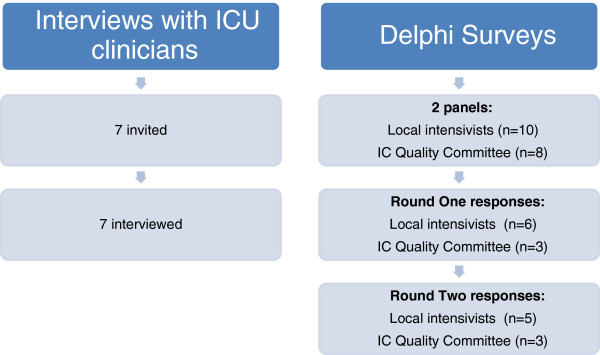
Participants and response rates.

The participants in each study component were ICU clinicians; seven were invited to participate in the semi-structured interviews, and 18 were included in the Delphi survey (see Figure 1). To answer the first study question, interviews were arranged with five intensivists, one clinical nurse consultant, and one research nurse at the ICU prior to commencing the Delphi study. Participants were selected based on their designation and role and had expressed an interest in quality and safety and improving care processes in their ICU. This served two purposes: 1) gathering relevant, multidisciplinary input; and 2) engaging local key stakeholders and potential clinical champions throughout the developmental and future implementation stages of the research project. Each person was contacted individually either in person or by telephone, a brief outline of the proposed discussion was provided, and following consent, a time to meet was arranged.

To answer the second study question, adequately represent the area under study, and maximize content validity, the expert panel invited to participate in the Delphi study comprised two sub-groups. First, members of a state-wide IC quality committee were invited: 1 state health department representative, 3 intensivists and 4 senior intensive care nurses (clinicians from various ICUs but none from the study ICU). All group members had extensive clinical experience in the specialty of intensive care, with an interest in quality and safety. Membership and participation in IC quality committee activities were voluntary. Second, all 10 intensivists from the ICU study site were also invited to participate. The study was discussed at an IC quality committee and local ICU management meeting respectively, to engage potential participants.

Background information pertaining to the Delphi component was presented at staff meetings attended by potential participants. Information included study context, purpose and methods, the role of the participant, and the process of the modified-Delphi technique. The importance of obtaining unbiased expert opinion that was to remain anonymous to other participants was highlighted. The information sheet, instructions and Delphi questionnaire were then circulated to potential participants by hardcopy (if in attendance at the meetings) or email. All participants were given a two week deadline to respond; on day 12 non-responders were sent personalized reminder emails in an attempt to gain maximum responses.

### Data collection instruments

In addition to exploring the relevance and adequacy of the process measures, the semi-structured interviews also sought identification of current issues and work practices that could impact on checklist content. The semi-structured approach to discussions with individuals enabled flexibility to obtain relevant information from the most appropriate person. All discussions commenced with the researcher providing general information pertaining to the proposed checklist intervention study (including proposed items derived from previous work) and how this pre-intervention study would help inform it. The remainder of the content differed depending on designation of the person and the following discussion points were covered:

● Intensivists (n = 5) – relevance and adequacy of proposed checklist items; opinions on inclusion or exclusion of checklist items for evaluation on the morning ward rounds, particularly those that were not explored in the medical records review (head-of-bed elevation for ventilated patients, pressure ulcer prevention, assessing responsiveness of sedated patients, checking the length of time since insertion for intravascular lines, review of antibiotic use and microbiology reports); current work practices and procedures that should be factored into checklist development and study design; and local policies and guidelines pertaining to potential checklist items.

● Clinical nurse consultant – current and planned unit initiatives; work practices associated with the planned checklist study and the proposed checklist items.

● Research nurse – current and planned research studies that may impact on the use and evaluation of the proposed process-of-care checklist.

The modified-Delphi Technique involved sufficient rounds in order to achieve consensus. For the initial round, participants were asked to rate a list of existing checklist statements generated from previous work, according to their clarity, conciseness and instructional value, on a 5-point likert scale (from strongly disagree to strongly agree). This modification to the traditional Delphi technique has been described as a ‘reactive Delphi’ as participants respond to previously prepared information, rather than generate items from scratch [20]. Participants were informed of checklist response options which included ‘clinical contraindication’. Additional space was provided for participants to make suggestions for improving each statement. Similar approaches have been used previously [13] with the qualitative comments section described as being a valuable addition to the questionnaire [21].

Statements that did not reach consensus were modified according to suggestions made by respondents. In the next round, two alternate statements for each component were devised – this served two purposes: 1) participant feedback on grouped responses; and 2) an opportunity to choose their preferred statement based on the refinements made after round one. Participants were asked to select which of the two statements they believed better described the process of care in terms of clarity, conciseness and instructional value. Additional space was provided for participants to make comments about the statements if required.

### Data management and analysis

Notes taken by the first author during interviews with clinicians were reviewed and important points that required consideration were listed. Key points were then discussed with the research team and were integrated into the first round of the Delphi questionnaire where appropriate.

*A-priori* decisions were made regarding the minimum standard for consensus agreement at the commencement of each Delphi round. For the first round, statements that obtained a median score of greater than or equal to 4.0 (representing ‘agree’ on the 5-point rating scale), had no ‘strongly disagree’ responses and no suggested changes, were accepted as having reached consensus. A similar scoring approach previously used a 4-point Likert scale [22], however in this instance it was decided that a neutral response option was required for respondents who had suggestions for improving the statements and neither agreed nor disagreed with a statement in its current form.

When devising the second round questionnaire, a decision was made to accept one of the two statements provided on the questionnaire that gained at least 51% of the respondents’ preference – an approach used previously [15,23,24]. Although this cut-off point has been questioned [25], there remains no scientific rationale or recognised guidelines for deciding appropriate consensus levels [26]. In addition to the majority vote, for a statement to have reached consensus there could not be a significant number of suggested changes.

After data collection was completed for each stage, data were de-identified prior to entry into a spreadsheet with identification numbers assigned to respondents. The participant log was kept separate from the data to be analyzed and password protected. To ensure credible interpretations, qualitative data were analyzed initially by the first author and then verified by the other authors. Conclusions based on qualitative data were discussed and agreed upon prior to further iterations of the Delphi questionnaire being developed and the reporting of results.

### Ethics

Human Research Ethics Committee (HREC) approval for this sub-study as part of a larger study program was obtained from Sydney-West Area Health Service & University of Technology, Sydney HRECs. Participants provided informed consent prior to involvement in this study.

## Results

Information garnered from interviews with seven ICU clinicians (outlined in Table 2) was used to develop checklist statements (see Table 3) for the first Delphi questionnaire. Although the target users of the checklist were established prior to these interviews (due to physician-lead morning ward rounds in the study ICU), the need for a physician-focused checklist was enhanced by the use of an existing nursing prompt card that outlined important daily care processes to be carried out by nursing staff. Discussion of this prompt card during the interviews leads to removal of the ‘sit out of bed’ and ‘pressure ulcer prevention’ items from the checklist (as detailed in Table 2).

**Table 2:** Issues identified by clinicians and how integrated into Delphi questionnaire

**Issue identified**	**Action**
Presence of policy documents on nutritional support, prevention of venous thromboembolism, prevention of upper gastrointestinal bleeding and need to align checklist items with these policies	Policy documents reviewed and factored into development of checklist statements to ensure consistency between the two
Sit out of bed managed by nursing staff and physiotherapists	Sit out of bed checklist item excluded
Checking the length of time since insertion of intravascular lines redundant due to unit policy (i.e. catheters left in place as long as clinically indicated), nursing prompt card (age of lines, dressings & site), & concurrent quality improvement project targeting improved insertion and care of central lines [27]	Checking the length of time since insertion of intravascular lines excluded
All medications should be reviewed on the morning round, not just antibiotics	Changed ‘review of antibiotics’ to ‘review of all medications’
Checking microbiology reports done in conjunction with the review of medications, so doesn’t need to be a separate item on the checklist	Checking microbiology reports excluded
Head-of-bed elevation for ventilated patients important to review by both medical and nursing – retain on checklist	Head-of-bed elevation retained
Assessing responsiveness of sedated patients an important aspect of medical rounds and needs to be retained	Assessing responsiveness of sedated patients retained
Pressure ulcer prevention managed by nursing staff, an item on the nursing prompt card	Pressure ulcer prevention excluded

**Table 3:** Checklist statements at each stage of the study

**After clinician interviews**	**After Delphi Round 1 (two alternate statements)**	**Final checklist statements (after Delphi Round 2)**
Nutritional plan has been implemented and/or reviewed (median = 5)	● Nutrition plan has been implemented and reviewed (43%)	Nutrition goals have been set and progress reviewed
	● Nutrition goals have been set and progress reviewed (57%)	
Pain has been assessed and is being managed (median = 5)	● Pain has been assessed and is being managed (37.5%)	Pain has been assessed, a management plan set and progress reviewed
	● Pain has been assessed, a management plan set and progress reviewed (62.5%)	
Sedation levels have been assessed and are being managed (median = 5)	● Sedation levels have been assessed and are being managed (86%)	Sedation target set, sedation level assessed and managed
	● Sedation levels have been assessed with target sedation score, a management plan is set and progress reviewed (14%)	
DVT prophylaxis is being delivered (median = 4)	● An appropriate means of delivering DVT prophylaxis has been chosen and is being delivered (43%)	Mechanical and/or drug DVT prophylaxis is being delivered
	● An appropriate means of delivering mechanical or pharmacological DVT prophylaxis has been chosen and is being delivered (57%)	
Head of the bed is raised 30–45 degrees (median = 4)	● Head of the bed is raised 30–45 degrees (37.5%)	Patient is positioned with the head of the bed raised >30 degrees
	● Head of the bed is raised greater than 30 degrees (62.5%)	
Stress ulcer prophylaxis is being delivered (median = 5)	Stress ulcer prophylaxis is being delivered (100%)	Stress ulcer prophylaxis is being delivered
Blood sugar level (BSL) is within defined limits for this patient or if outside limits is being treated (median = 5)	● BSL is within defined limits for this patient or if outside limits are being treated (62.5%)	BSL limits have been set and are being managed to achieve those limits
	● Blood glucose limits have been defined, BSL is within defined limits or if outside limits are being treated (37.5%)	
Patient’s readiness to extubate has been assessed (median = 5)	● Patient’s readiness to be weaned from mechanical ventilation has been assessed (71%)	Patient’s readiness to be weaned from mechanical ventilation has been assessed
	● Ability of the patient to weaned from mechanical ventilation has been assessed and a ventilation plan has been set (29%)	
All medications have been checked and reviewed (median = 5)	● All medications have been checked and reviewed (71%)	All medications have been checked and reviewed
	● Indications and dosing documentation for all current medications reviewed and correct (29%)	

Other than broadening one of the checklist items i.e. from reviewing antibiotics to reviewing all medications, no further additions to the checklist items were suggested. Importantly, other than the issues identified in Table 2, participants believed the proposed checklist items adequately covered important elements of care to be checked for each patient on the morning ward rounds, and were applicable to standard or expected clinical practice in the ICU. Three intensivists noted that some checklist items were also supported by local policies and guidelines i.e. nutrition [28], DVT prophylaxis [29], and stress ulcer prophylaxis [30], and expressed the need to ensure checklist statements were consistent with existing policy documents.

In Round 1 of the Delphi survey, a total of 9 (56%) responses were received (see Figure 1). All statements achieved a median ≥ 4.0; equivalent to ‘agree’ and ‘strongly agree’ and there were no ‘strongly disagree’ responses (see Table 4). Suggestions were provided for changing the wording for all statements, except for stress ulcer prevention. All comments were considered and where appropriate, integrated into two alternate statements for each remaining care component for the expert panel’s consideration in the second round of the Delphi process (Table 3 illustrates the evolution of checklist statements over the course of the study). As an example, although 89% of respondents either agreed or strongly agreed with the statement for pain, 11% neither agreed nor disagreed and a more detailed statement was suggested. For the next Delphi round, the original statement was provided along with an alternate version to gauge the majority preference.

**Table 4:** Descriptive statistics for first round Delphi survey responses by care component

**Care component**	**Median [IQR]**	**Mode [Min – Max]**	**% agree & strongly agree**
Stress ulcer prevention	5 [4–5]	5 [4–5]	100
Pain	5 [4–5]	5 [3–5]	89
Head-of-bed elevation	4 [4–5]	4,5 [3–5]	89
Medications	5 [3.5–5]	5 [3–5]	78
Sedation	5 [3.5–5]	5 [2–5]	78
Glucose management	5 [3.5–5]	5 [2–5]	78
Nutrition	5 [3–5]	5 [2–5]	78
Readiness to wean from mech vent	5 [3–5]	5 [2–5]	78
DVT prophylaxis	4 [3.5–5]	4 [2–5]	78

*IQR* inter-quartile range, *Min* minimum, *Max* maximum, *Mech vent* mechanical ventilation. Responses were scored 1 = strongly disagree; 2 = disagree; 3 = neither agree nor disagree; 4 = agree; 5 = strongly agree.

In Round 2, 8 (50%) responses were received (see Figure 1). For each item, statements with the majority (>50%) of preferences was either accepted or slightly amended in response to suggestions for further changes to the statement. Three statements were accepted without the need for further changes – nutrition (57%), extubation (71%), medications (71%). The remaining five statements (pain 62.5%; sedation 86%; DVT prophylaxis 57%; head-of-bed elevation 62.5%; glucose 57%) required only minor adjustments to wording to be clearer, more concise, and to improve instructional value. The few comments made by experts in this round improved the statements without changing the context or key message e.g. abbreviating ‘An appropriate means of delivering mechanical or pharmacological DVT prophylaxis has been chosen and is being delivered’ to ‘Mechanical and/or drug DVT prophylaxis is being delivered’.

Following this round, it was evident that no further rounds were required as there was sufficient coherence in participants’ responses. Following recommendations for checklist composition [1], minor editorial changes ensured that terminology and phrasing was consistent across all nine checklist statements, which were purposely ordered to align with the FASTHUG mnemonic [31]. The resulting final checklist statements are outlined in Table 3.

## Discussion

The key outcomes of this study were the development and validation of a suite of clear and concise statements on nine essential processes of care, to be used as a checklist for supporting practice during daily rounds in an ICU. Study findings added evidence in support of the content validity of the checklist items - particularly the relevance, adequacy, and clarity of checklist statements.

Interviews with local ICU clinicians confirmed the adequacy of content covered by the process-of-care checklist as well as providing initial information pertaining to the practice relevance of each individual statement. These informants also offered important information on the local context, which supported the refinement of checklist statements for inclusion in the first round Delphi survey. These initial revisions provided additional credibility to the Delphi process by ensuring the preliminary statements were relevant to the local ICU.

The modified-Delphi technique used was developed in line with contemporary research guidelines [12,32] to address the limitations of other research in this field and enhance rigor in this type of study. This was exemplified by the methods used (i.e. incorporation of information obtained from a literature review [3], a point-prevalence study [6], and a criterion-related validation study) prior to and during the pre-Delphi interviews that consequently informed revision of the checklist items. This preliminary information was then incorporated into the first Delphi round, as this approach may be more reliable than an open-first round Delphi survey [12].

Only two rounds were required to reach consensus. When viewed collectively, the findings from both Delphi rounds demonstrated the “stability” of responses, suggesting a reasonable indicator of consensus [25,32]. Despite almost gaining consensus after the first round, several suggestions were made to improve the clarity for all but one of the statements. After second round responses were collated, all statements had either been accepted without further changes, or suggestions for changes had been integrated into the final statements. This is evidence of: 1) previous work on developing checklist content was a sufficient starting point for this modified-Delphi study; 2) only refinements to the existing statements were required to generate the most clear, concise and instructive statements. It is likely that the preliminary work also ensured quick replies from panel respondents and a shorter time to reach consensus. Other studies, particularly those that generated content from scratch reported much longer study periods [13,26].

Although there is contention pertaining to acceptable consensus levels, recent recommendations suggested that levels be: established prior to data collection; based on the importance of the research topic; and supported by rational justification [26]. The decision to accept second-round statements with at least 51% agreement was based on the following: 1) majority agreement was more practicable than 100% consensus given there could be countless minor variations of the same statement that met the criteria of being clear, concise and instructive statements; 2) there was near-consensus after the first survey round; and 3) to minimize respondent burden and exhaustion from busy ICU clinicians, and managers, which has also been reported [20].

Purposive sampling for the Delphi study allowed the selection of experts best able to provide advice on statement development. Similar to a previous study [15], the use of two expert panels strengthened the validation process. The participation of IC quality committee members lent support to the external validity of the checklist statements i.e. they can be used in all general ICUs as a starting point from which local clinician input can be obtained. The panel of intensivists provided the desired local ICU input, ensuring that terminology was applicable for use in that ICU. Their involvement enabled an opportunity for input into tool development that would be used by themselves or their colleagues in routine practice, and also facilitated engagement in planned future studies.

The Delphi panel size of at least 8 respondents was in line with recommendations that the membership number be relevant to the purpose of the study, the selected design, and data collection time frame [12,13]. The panel size was also large enough to obtain a substantial amount of useful feedback, and proved adequate for reaching consensus on the wording of checklist statements. A larger sample size may have generated more variations that still met the criteria of being clear, concise and instructive, but this may have prolonged the process unnecessarily, and may have diminished applicability of the statements to the local setting. Similar to other studies using the Delphi technique, treatment of data obtained from panel members was de-identified (i.e. individual responses were not made available to other participants), removing any risk of influence on group conformity, power, and the effect of others on responses [19].

Unlike previous studies [16-18], results of the Delphi technique were reported for each round, with key suggestions for improvements to the statements reflected in the second round Delphi questionnaire and the final checklist statements. The importance of describing the sampling process in detail has also been emphasized in the literature [32], and as such, detailed information pertaining to the selection processes and characteristics of the panel members has been reported. This level of data collection and reporting allows for increased transparency for the purposes of study replication and provides evidence of sufficient methodological rigor in developing the checklist statements.

### Limitations

There were limitations to this study. First, the response rate to the Delphi survey was moderate (56% and 50% in the two rounds, respectively). Non-responders were not followed-up further and therefore their reasons for non-participation were unknown. A previous paper [15] reported a range of response rates from two Delphi studies – the highest response rate was 73% for a second-round Delphi survey of 22 international panelists; the lowest was 39% for a second-round survey of 18 regional panelists. They partly attributed the higher response to pre-selecting panelists that indicated their willingness to participate (which was not the case with other panels they used), resulting in a motivated, committed panel of experts. Response rates to our study were similar to a first-round survey of one of these previous studies [15] (i.e. 56% of 16 national panelists), supporting the notion of pre-selecting willing participants as a possible solution to improve response rates.

Where possible, other suggestions made in the literature for obtaining an optimal response rate were followed, including: making personal contact and building rapport by informing participants to enhance personal ownership of the project [26]; and planned follow-up [33] in the form of a reminder email. Similar to earlier studies [15], we opted not to pursue non-responders further as we did not wish to pressure already busy clinicians who we needed to be supportive of planned studies that required their contribution. It was possible however, that non-responders were not interested in checklist development, did not have anything to add to the process, or were not able to make study participation a priority given their primary role was in clinical and teaching responsibilities.

Participants within each of the panels were known to each other and all participants were known to the researcher. The risk of potential bias was minimized by allowing respondents to complete the questionnaires in their own time, ensuring responses remained strictly anonymous within the Delphi process, and providing synthesized feedback during the second-round survey. It has been suggested that this kind of ‘quasi-anonymity’ could actually motivate panelists to participate, discourage ill-considered hasty judgments, and ensure some level of accountability for the responses given [20].

Due to practical constraints of creating a parallel-form measure we did not test reliability by comparing the final checklist statements generated using the Delphi technique with statements generated by another method of developing the tool – for example via focus groups or consensus meetings with experts [12]. Coordinating a single meeting time to suit all experts would have been difficult, particularly since the majority had clinical duties. Even if one had been arranged, it is questionable whether a group meeting can produce reliable results given the risk of bias with group conformity [19].

### Recommendations for research

There are a few key areas that require evaluation in future studies. First, it is important to verify these findings with further research conducted in clinical settings. The checklist items generated should be evaluated for their practical use, interpretation and clinical utility. Second, testing the reliability of items should be undertaken to establish whether the items produce consistent results. Third, the methods used for checklist development and validation also have applicability beyond the ICU and can be tested as a model for improvement in other clinical areas irrespective of geographical location.

## Conclusion

The use of both interviews and a modified-Delphi technique with ICU clinicians produced a series of checklist items that represented relevant content for essential practices in the process-of-care for ICU patients, and were deemed clear, concise, and instructive statements for use by intensivists during the morning clinical rounds. The use of rigorous methods lends support to the content validity of the process-of-care checklist which was to be used as the intervention in a prospective research study conducted in the same ICU. Transparent reporting of both methods and results allow for study replication and further testing for the purposes of determining reliability and clinical utility.

## Abbreviations

BSL: Blood sugar levels; DVT: Deep vein thrombosis; FASTHUG: Feeding Analgesia Sedation Thromboembolism prophylaxis Head-of-bed elevation stress Ulcer prevention Glucose control; HREC: Human Research Ethics Committee; IC: Intensive Care Unit; IC: Intensive Care.

## Competing interests

The authors declare that they have no competing interests.

## Authors’ contributions

Study design: DE, KC; data collection: KC; data analysis: KC, DE, AB; manuscript preparation: KC; critical revision of manuscript: DE, AB, KC. All authors read and approved the final manuscript.

## Pre-publication history

The pre-publication history for this paper can be accessed here:

http://www.biomedcentral.com/1472-6963/13/380/prepub
